# Accuracy of Colposcopically Directed Biopsy: Results from an Online Quality Assurance Programme for Colposcopy in a Population-Based Cervical Screening Setting in Italy

**DOI:** 10.1155/2015/614035

**Published:** 2015-06-09

**Authors:** Mario Sideri, Paola Garutti, Silvano Costa, Paolo Cristiani, Patrizia Schincaglia, Priscilla Sassoli de Bianchi, Carlo Naldoni, Lauro Bucchi

**Affiliations:** ^1^Preventive Gynaecology Unit, European Institute of Oncology, Via Giuseppe Ripamonti 435, 20141 Milan, Italy; ^2^Department of Obstetrics and Gynaecology, University Hospital, Via Aldo Moro 8, Cona, 44124 Ferrara, Italy; ^3^Department of Obstetrics and Gynaecology, St. Orsola Hospital, Via Giuseppe Massarenti 9, 40138 Bologna, Italy; ^4^Cervical Cancer Screening Unit, Bologna Health Care District, Via della Repubblica 11, San Lazzaro di Savena, 40068 Bologna, Italy; ^5^Cancer Prevention Centre, Ravenna Health Care District, Viale Vincenzo Randi 5, 48121 Ravenna, Italy; ^6^Department of Health, Regione Emilia-Romagna, Viale Aldo Moro 21, 40127 Bologna, Italy; ^7^Romagna Cancer Registry, Romagna Cancer Institute (IRST) IRCCS, Via Piero Maroncelli 40, Meldola, 47014 Forlì, Italy

## Abstract

*Purpose*. To report the accuracy of colposcopically directed biopsy in an internet-based colposcopy quality assurance programme in northern Italy.* Methods*. A web application was made accessible on the website of the regional Administration. Fifty-nine colposcopists out of the registered 65 logged in, viewed a posted set of 50 digital colpophotographs, classified them for colposcopic impression and need for biopsy, and indicated the most appropriate site for biopsy with a left-button mouse click on the image.* Results*. Total biopsy failure rate, comprising both nonbiopsy and incorrect selection of biopsy site, was 0.20 in CIN1, 0.11 in CIN2, 0.09 in CIN3, and 0.02 in carcinoma. Errors in the selection of biopsy site were stable between 0.08 and 0.09 in the three grades of CIN while decreasing to 0.01 in carcinoma. In multivariate analysis, the risk of incorrect selection of biopsy site was 1.97 for CIN2, 2.52 for CIN3, and 0.29 for carcinoma versus CIN1.* Conclusions*. Although total biopsy failure rate decreased regularly with increasing severity of histological diagnosis, the rate of incorrect selection of biopsy site was stable up to CIN3. In multivariate analysis, CIN2 and CIN3 had an independently increased risk of incorrect selection of biopsy site.

## 1. Introduction

Colposcopy aims at detecting macroscopic changes in colour and morphology of cervical mucosa. Comparison of these features with established patterns of disease allows classifying the observed lesions and identifying abnormal areas that warrant biopsy.

The colposcopic impression of any abnormality, however, is prone to observer variation. This is potentially associated with a low inter- and intraobserver agreement in interpretation of colposcopic abnormalities and with a low accuracy of colposcopically directed biopsy in defining extent and severity of lesions. Several cross-sectional and prospective studies published between the 1990s [1, 2] and the last decade [3–6] have cast doubt on the effectiveness of biopsy in detecting the presence of high-grade cervical intraepithelial neoplasia (CIN).

Low sensitivity for detection of high-grade disease may have serious clinical consequences. In particular, it may cause early invasive lesions to be inadvertently treated by an ablative technique [2, 7]. Disease relapse, which has been described in these patients [8], may erode the clinicians' confidence about conservative treatments. Nondiagnosis of carcinoma flaws quality control procedures for cytology [9] and invalidates the clinical studies of preinvasive disease that use biopsy as a gold standard [1].

These problems are complicated by the insufficient diffusion of quality assurance (QA) programmes for colposcopy. These programmes that should be based on interactive retraining sessions and large agreement and accuracy studies would allow identifying specific areas of improvement, selecting a set of well-defined and highly reproducible colposcopic features of cervical abnormalities, and increasing the colposcopists' competence as well as the appropriateness of their clinical decisions.

In the first session of a permanent online colposcopy QA programme that is being conducted in Italy, the participants evaluated a test set of digital colpophotographs. The current article reports an analysis of the correctness of their decisions for biopsy.

## 2. Materials and Methods

### 2.1. Setting

The population-based, triennial Pap smear screening service that covers women aged 25–64 years living in the Emilia-Romagna Region of northern Italy is described elsewhere [10]. Colposcopy assessment for women with abnormal screening results is carried out by specially appointed gynaecologists and gynaecologist oncologists. Over the past decade, the colposcopists working in the screening centres have been targeted by several on-site colposcopy QA initiatives. In 2009-2010, an internet-based QA programme was developed.

### 2.2. Design

A detailed protocol of the programme can be found and free-accessed elsewhere [11]. In brief, a log-in web application was created and made accessible on the website of the regional Administration. Between December 2010 and February 2011, the 65 screening colposcopists were invited to participate on a voluntary basis. Fifty-nine registered, logged-in, viewed a posted set of 50 colpophotographs selected by an expert committee, and classified them according to colposcopic impression, visibility of the squamocolumnar junction, and need for biopsy. The images were accompanied by a caption with information about patient age, last Pap smear result, and human papillomavirus test result (if any). The participants indicated the single most appropriate site for biopsy with a left-button mouse click on the image. This site was automatically checked against an area identified by the committee as the most appropriate one from which to take a sample. The size and shape of the area varied according to its colposcopic appearance. Its coordinates were mapped inside the source code of the software. The site selected by the colposcopists was automatically classified into correct and incorrect. After completing the test, they received online a set of personal results. The programme had no administrative functions (ranking, accreditation, etc.).

The committee classified the colposcopic impression and identified the single most appropriate biopsy site with a joint discussion. Original histological information, including normal histology and biopsy not performed, was known to the selectors but was not assumed to represent a gold standard for the colposcopic impression and the need of a biopsy [11].

### 2.3. Colpophotographs

Technical details of acquisition of the test set of images can be found elsewhere [11]. In brief, 250 high-definition digital colpophotographs were obtained from women with abnormal Pap smear results consecutively attending two screening centres randomly selected out of the total 11 centres. From this basic set, 50 images were selected based on the following criteria: they were well-representative of major normal and abnormal colposcopic findings; the cervix was entirely visible; there were no light reflections, colour artifacts, shaded areas, or mucus accumulation; and the patient had not been treated previously. The rationale for these criteria is discussed elsewhere [11].

### 2.4. Classification of Colposcopic Impression

Colposcopic impression was classified as negative; abnormal, grade 1 (G1); abnormal, grade 2 (G2); and suspected invasive cancer (Cancer). These categories were equivalent to the colposcopic patterns that the International Federation for Cervical Pathology and Colposcopy classification of 2002 [12] designated as normal colposcopic findings; abnormal colposcopic findings, minor changes; abnormal colposcopic findings, major changes; and colposcopic features suggestive of invasive cancer.

### 2.5. Rationale and Objectives of the Current Study

In May 2011, a plenary seminar was organized to discuss the overall results and to perform an interactive review of the test set of images. An article reporting agreement data on colposcopic impression has recently been published [13].

The rationale of the study that is presented here has been described in detail elsewhere [11]. In brief, although the committee did not consider the original histological diagnosis as a gold standard, comparing the interpretation of colposcopic findings and the decision for biopsy with the histological diagnosis of the underlying lesion was nevertheless important in that it provided an approximate measure of the probability for women with abnormal Pap smear results to receive a false-negative or false-positive colposcopy assessment.

In particular, the current study was undertaken to determine the probability of a patient with abnormal colposcopic findings and a histologically confirmed cervical lesion not having biopsy or having biopsy in an incorrect cervical site. We evaluated (1) the nonbiopsy rate and the rate of incorrect selection of biopsy site according to colposcopic impression formulated by the committee; (2) the nonbiopsy rate and the rate of incorrect selection of biopsy site according to original histological diagnosis; and (3) the association of patient characteristics and colposcopist characteristics with the probability of biopsy failure of both types.

### 2.6. Data Analysis

Data analysis was based on a total of 2950 paired colposcopist-committee observations resulting from the product of 59 colposcopists and 50 images.

Ninety-five percent confidence intervals (CI) around rates were calculated according to standard methods [14].

In the analysis of factors associated with biopsy failures, all variables were treated as categorical. The patient age and colposcopist age were dichotomized by the median values. The chi-square test for heterogeneity and trend was used to estimate the strength of univariate associations. A *P* value < 0.05 was considered statistically significant. Multivariate analysis was performed using a multiple logistic regression model (backward stepwise selection). The level for removal of variables was set at *P* = 0.10. An odds ratio with a 95% CI that did not include the unity was considered statistically significant.

## 3. Results

### 3.1. Nonbiopsy and Incorrect Selection of Biopsy Site by Colposcopic Impression

Table 1 shows the colposcopists' performance according to the colposcopic impression formulated by the committee. Overall, the colposcopists considered biopsy to be indicated more often than the committee, that is, in 2071 of 2950 instances versus 1947 (rate, 0.70 versus 0.66; ratio, 1.06; 95% CI, 1.00 to 1.13). This was entirely explained by the fact that they opted for biopsy in 20% of cases interpreted to be negative (and thus unworthy of further investigations) by the committee. Conversely, biopsy was omitted in about 10% of G1 changes, and 1% of G2 changes. No such cases were observed when the committee formulated the impression of Cancer. As far as the biopsy site is concerned, the rate of errors was stable at about 0.10 in both G1 and G2 and decreased to 0.01 in Cancer. Total biopsy failure rate, which peaked at 0.21 in G1, decreased regularly to 0.09 in G2 and 0.01 in Cancer.

### 3.2. Nonbiopsy and Incorrect Selection of Biopsy Site by Original Histological Diagnosis

Table 2 shows the frequency of nonbiopsy and incorrect selection of biopsy site according to original histological diagnosis. The pattern of results was closely similar to that in Table 1. Total biopsy failure rate was 0.20 in CIN1 and then decreased to approximately 0.10 in CIN2 and CIN3/AIS and 0.02 in carcinoma. However, the decreasing trend was more rapid for nonbiopsy rate, while errors in the selection of biopsy site were stable in all grades of CIN and decreased only in carcinoma. For this reason, they were the majority of total biopsy failures (133/197 or 68%), and this was entirely accounted for by their greater proportion in CIN2 and CIN3.

### 3.3. Factors Associated with Incorrect Selection of Biopsy Site

On account of the above finding, analysis of factors associated with biopsy failures was restricted to incorrect selection of site. Results are shown in Table 3. In univariate analysis, the probability of biopsy site being incorrectly selected decreased with increasing severity of the colposcopic impression and of histological diagnosis and was greater when the squamocolumnar junction was not, or not entirely, visible. In multivariate analysis, these associations remained statistically significant. In particular, using CIN1 as a reference category, the adjusted odds ratio for incorrect selection of biopsy site was approximately between 2 and 2.5 in CIN2 and CIN3 while dropping to about 0.30 in carcinoma.

## 4. Discussion

### 4.1. Rationale Issues

Colposcopically directed punch biopsy is affected by well-known biases that arise from colposcopic pattern recognition and from collection, processing, and reporting of biopsy samples. In addition to this, it is increasingly understood that the colposcopic pattern is an independent risk stratifier in the patient management algorithm, which includes many pieces of clinical information. Moreover, biopsy in medicine is typically used to confirm the diagnosis of a suspected condition, whereas assessment of precancerous cervical disease is often done with the excision of the entire lesion.

Despite these changing concepts, however, assessment of cervical disease status still relies in most instances on the histological report of colposcopically directed punch biopsy (or biopsies), which remains a critical step in the management of women with abnormal Pap smear results.

### 4.2. Test Conditions

We have previously discussed the methodological problems involved in colposcopy QA and, thus, in our own programme [11, 13]. The basic problem is that the test was conducted under artificial conditions. This facilitated the recognition of colposcopic features and the accuracy in indicating the need for biopsy and in selecting the place from which to take the sample. Opposite biases also existed, such as the impossibility of increasing the magnification of tissues. It appears that the overall sensitivity of the diagnostic process cannot be directly inferred from the sensitivity of colposcopically guided biopsy in a QA environment.

In addition, the participants were allowed a single opportunity to choose the biopsy site. This is different from the clinical real-world situation, although it enabled them to receive a direct feedback of the correctness of the chosen site.

### 4.3. Design

Correlating the colposcopic interpretation and the decision for biopsy with the histological diagnosis provides an approximate estimate of the probability of a false-negative or false-positive colposcopy assessment [11, 13]. The problem with this approach is that there is no absolute histological gold standard on which to rely. This can be established by cone biopsy or loop excision biopsy [2, 6, 7], by biopsy of colposcopically detected abnormalities plus random biopsies from normal-appearing quadrants [4] or by endocervical curettage plus random biopsies from normal-appearing areas [15].

The current study was not comparable with these designs. We used virtual substrates and we made the unproven assumption that the biopsy site chosen by the committee was on the worst-looking area. Moreover, given the relative subjectivity of the colposcopic impression, a single-shot biopsy decreased the chance of selecting the area with higher-grade colposcopic abnormalities and did not exclude the possibility that foci of severe squamous lesions could be found in specimens taken from areas with minor colposcopic changes.

### 4.4. Histological Diagnosis

The patients whose colpophotographs and data were used in the current study were originally diagnosed in two screening centres that follow certified QA procedures including those for cytology and histology in cancer screening [16]. These facts notwithstanding our assumption that the original histological diagnosis reflected the actual state of disease are unwarranted. It may have occurred, for example, that quality and amount of biopsied tissue were insufficient and that the pathologist's reporting was inaccurate.

However, our results must be viewed from the perspective that misclassifications do not create an association between two variables. Rather, they weaken or abolish an association if it exists. Following this line of reasoning, we can safely assume that the observed steep decrease in total biopsy failure rate from CIN1 to carcinoma was not generated by a misclassification bias.

### 4.5. Interpretation of Results

Due to the above considerations, extrapolation of our results to a field situation as well as external comparisons with other studies should be done with caution. Conversely, internal comparisons are free of biases resulting from test conditions. From this perspective, some findings deserve attention. First, overall biopsy rate was higher among the participating colposcopists compared with the expert committee, reflecting a higher level of diagnostic uncertainties. Specifically, among colposcopists with limited experience, a non-conservative approach to biopsy is positively associated with the probability of disease detection [17–19].

Second, total biopsy failure rate decreased steadily with increasing histological severity of the lesion. This is explained by our previous finding of a strong correlation between colposcopic impression and original histological diagnosis [13]. The correlation between the visual aspects of the cervix and the severity of the underlying epithelial changes is imperfect [15, 18] but not weak.

Third, incorrect selection of biopsy site was the most common type of biopsy failure, and this was entirely due to the fact that it was more frequent than nonperformance of biopsy in CIN2 and CIN3. In multivariate analysis, after adjustment for the colposcopic impression and the visibility of the squamocolumnar junction, the risk of incorrect selection of biopsy site was confirmed to be significantly increased in CIN2 and CIN3 and extremely low in carcinoma.

Problems with biopsy site in high-grade CIN are difficult to interpret. Errors in selecting a biopsy site in a large surface lesion have often been postulated to explain nondiagnosis of CIN3 [20] and microinvasive carcinoma [21]. Complex atypical areas of the transformation zone including central small high-grade foci and an external large low-grade lesion may be incorrectly interpreted by colposcopists. Probably, lesions of this type are most commonly encountered in a regularly repeated screening setting because they are of recent onset and at an early stage of development.

In any case, our finding provides another confirmation that the concerns regarding the sensitivity of biopsy for high-grade CIN are justified [1–6]. Several potential solutions to this problem are under consideration. Some options that are being proposed include increasing the number of biopsy specimens [17], that is, taking multiple biopsies from the worst-looking lesion and other abnormal areas and taking random biopsies from all normal-appearing areas [18]. The latter approach is particularly controversial.

Several factors have been hypothesised to influence the accuracy of biopsy. The level of supporting evidences, however, is low [7]. Two important, although expected, findings of our study were that the risk of incorrect selection of biopsy site decreased steadily with increasing severity of the colposcopic impression and that it was greater when the squamocolumnar junction was not, or not entirely, visible.

### 4.6. Conclusions

Although the existence of a problem with selection of biopsy site specifically in high-grade CIN was confirmed, total biopsy failure rate decreased with increasing severity of both colposcopic impression and histological diagnosis and was almost nil for invasive carcinoma. Before undertaking aggressive biopsy strategies aimed at increasing the number of specimens, an attempt should be made to improve the detectability of high-grade CIN through large training programmes.

## Figures and Tables

**Table 1:** Nonbiopsy and incorrect selection of biopsy site according to the colposcopic impression formulated by the committee.

	Committee decision on biopsy according to colposcopic impression					Total
	Biopsy: no (negative)	Biopsy: yes				
		G1	G2	Cancer	Subtotal	
Colposcopist decision on biopsy						
No	803	64	12	0	76	879
Yes, incorrect site	NA	58	85	2	145	145
Yes, correct site	NA	468	1024	234	1726	1726
Yes, subtotal	200	526	1109	236	1871	2071
Total	**1003**	**590**	**1121**	**236**	**1947**	**2950**
Nonbiopsy rate	NA	0.11 (0.08–0.13)	0.01 (0.01-0.02)	0.00 (0.00–0.02)	0.04 (0.03–0.05)	NA
Incorrect biopsy site rate	NA	0.10 (0.08–0.12)	0.08 (0.06–0.09)	0.01 (0.00–0.03)	0.07 (0.06–0.09)	NA
Total biopsy failure rate	**NA**	**0.21 (0.17–0.24)**	**0.09 (0.07–0.11)**	**0.01 (0.00–0.03)**	**0.11 (0.10–0.13)**	**NA**

G1: abnormal, grade 1; G2: abnormal, grade 2; Cancer: suspected invasive cancer.

**Table 2:** Nonbiopsy and incorrect selection of biopsy site according to original histological diagnosis.

	CIN1^a^	CIN2	CIN3/AIS	Carcinoma	Total
Colposcopist decision on biopsy					
No	48	5	9	2	64
Yes, incorrect site	35	21	75	2	133
Yes, correct site	330	210	860	232	1632
Yes, subtotal	365	231	935	234	1765
Total	**413**	**236**	**944**	**236**	**1829**
Nonbiopsy rate	0.12 (0.09–0.15)	0.02 (0.01–0.05)	0.01 (0.00–0.02)	0.01 (0.00–0.03)	0.03 (0.03-0.04)
Incorrect biopsy site rate	0.08 (0.06–0.12)	0.09 (0.06–0.13)	0.08 (0.06–0.10)	0.01 (0.00–0.03)	0.07 (0.06–0.09)
Total biopsy failure rate	**0.20 (0.16–0.24)**	**0.11 (0.07–0.15)**	**0.09 (0.07–0.11)**	**0.02 (0.00–0.04)**	**0.11 (0.09–0.12)**

CIN: cervical intraepithelial neoplasia; AIS: adenocarcinoma in situ.

^a^Fifty-nine CIN1 cases were excluded from evaluation because the committee did not consider them worthy of biopsy based on colposcopic findings and, thus, did not classify the correctness of biopsy site as indicated (if any) by the colposcopists.

**Table 3:** Factors associated with the odds ratio for incorrect selection of biopsy site (total number of observations 1829).

Factor^a^	Number of observations	Number (%) with incorrect biopsy site	*P*	Univariate odds ratio (95% CI)	Multivariate odds ratio (95% CI)^b^
Colposcopic impression					
Negative	NA	NA	0.000		
G1	472	46 (9.7)		1.00 (referent)	1.00 (referent)
G2	1121	85 (7.6)		0.76 (0.52–1.11)	0.46 (0.27–0.80)
Cancer	236	2 (0.8)		0.08 (0.02–0.33)	0.09 (0.02–0.42)
Squamocolumnar junction					
Visible	1416	85 (6.0)	0.000	1.00 (referent)	1.00 (referent)
Not, or not entirely, visible	413	48 (11.6)		2.06 (1.42–2.99)	2.46 (1.62–3.73)
Original histological diagnosis					
CIN1	413	35 (8.5)	0.001	1.00 (referent)	1.00 (referent)
CIN2	236	21 (8.9)		1.05 (0.60–1.86)	1.97 (1.01–3.85)
CIN3/AIS	944	75 (7.9)		0.93 (0.61–1.42)	2.52 (1.32–4.80)
Invasive carcinoma	236	2 (0.8)		0.09 (0.02–0.39)	0.29 (0.06–1.34)

G1: abnormal, grade 1; G2: abnormal, grade 2; Cancer: suspected invasive cancer; CIN: cervical intraepithelial neoplasia; AIS: adenocarcinoma in situ; CI: confidence interval; NA: not applicable.

^a^Patient age, colposcopist age, and participation in previous local quality assurance initiatives were not associated with incorrect selection of biopsy site in univariate analysis (*P* > 0.05) and were not included in logistic regression analysis.

^b^From a multiple logistic regression model with backward stepwise selection of variables. The level for removal of variables was set at *P* = 0.10.
